# Enhanced expression of miR-21 and miR-150 is a feature of anti-mitochondrial antibody-negative primary biliary cholangitis

**DOI:** 10.1186/s10020-019-0130-1

**Published:** 2020-01-16

**Authors:** Urszula Wasik, Agnieszka Kempinska-Podhorodecka, Dimitrios P. Bogdanos, Piotr Milkiewicz, Malgorzata Milkiewicz

**Affiliations:** 1 Department of Medical Biology, Pomeranian Medical University, Szczecin, Poland; 2 Department of Rheumatology and Clinical Immunology, University of Thessaly, Larissa, Greece; 3 Translational Medicine Group, Pomeranian Medical University, Szczecin, Poland; 4 Liver and Internal Medicine Unit, Medical University of Warsaw, Warsaw, Poland

**Keywords:** Primary biliary cholangitis, AMA, miR-21, miR-150

## Abstract

**Background & Aims:**

Anti-mitochondrial-autoantibodies (AMA) remain a hallmark of Primary Biliary Cholangitis (PBC) however approximately 10% of patients test negative for these antibodies. They do not differ in terms of biochemistry or clinical presentation from AMA positive ones. Epigenetics play a key role in immune signalling. Two microRNAs (miRs), namely, miR-21 and miR-150 are known to be involved in liver inflammation and fibrosis. The expression of those two microRNAs and their downstream targets were analyze in the context of AMA-status and the stage of liver fibrosis.

**Methods:**

The relative levels of miR-21 and miR-150 and their target genes: cMyb, RAS-guanyl-releasing protein-1(RASGRP1), and DNA-methyltransferase-1(DNMT1) were determined by Real-Time PCR in serum, liver tissue and peripheral blood mononuclear cells (PBMCs) of patients with PBC.

**Results:**

Serum expressions of miR-21 and miR-150 were significantly enhanced in AMA-negative patients, and they inversely correlated with disease-specific AMA titers in PBS patients. In PBMCs, an increased expression of miR-21 correlated with decreased levels of RASGRP1 and DNMT1 mRNAs whereas, the level of miR-150 remained comparable to controls; and cMyb mRNA was downregulated. In cirrhotic livers, the level of miR-21 was unchanged while miR-150 expression was increased.

**Conclusion:**

This study convincingly report, that AMA-negative PBC is characterized by notable alternations of miR-21 and miR-150 and their downstream targets compared to AMA-positive patients underlining their possible importance in the induction of the disease and its progression to fibrosis.

## Introduction

Primary biliary cholangitis (PBC) is a chronic, autoimmune, cholestatic liver disease that may lead to liver failure. The diagnostic hallmark of PBC is the presence of anti-mitochondrial antibodies (AMA) which is present in 90% of the patients. Affected patients with AMA-negative PBC appear to have more advanced bile duct damage and worst prognosis than AMA positive for reasons poorly understood (Juliusson et al. 2016). Despite the documented lack of an autoantigen-driven mechanism in AMA-negative PBC, as in the case of the AMA-positive form, the disease is caused by the induction of autoreactive response which leads to the destruction of biliary epithelial cells of the small and medium-size intrahepatic bile ducts. Progression of PBC is largely sustained by an ongoing activation of autoantigen-specific B cells in blood and the expansion of liver-resident autoantigen specific CD4^+^ T cells, CD8^+^ T cells (as well as NK and NKT cells) which ultimately causes bile duct injury (Bogdanos et al. 2013).

As in other autoimmune diseases, valuable information on PBC immunopathology can be obtained by the study of microRNAs (miRNAs). MicroRNAs is a family of small non-coding RNAs, that regulate expression of genes at the post-transcriptional level. Up till now, a variety of miRNAs have been recognized as key players in orchestrating immune responses, including miR-21 and miR-150. MiR-21 modulates T cell activation and apoptosis, Th17 cell differentiation, Treg cell development and Th1/Th2 balance (Lu et al. 2011; Murugaiyan et al. 2015). In immune system, miR-21 acts via downregulation of RAS guanyl-releasing protein 1 (RASGRP1), a protein that link TCR signal transduction to Ras and MAPK activation. RASGRP1 inhibition promotes cell hypomethylation via suppression of DNA methyltransferase 1 (DNMT1), followed by overexpression of autoimmune-associated methylation-sensitive genes (Pan et al. 2010). Hence, it is not surprising that increased miR-21 expression is closely associated with the maintenance of autoreactive immunity (Stagakis et al. 2011). In dominant-negative TGF-β receptor II mice, which spontaneously develop an autoimmune cholangitis similar to human PBC the T cell dysregulation is associated with upregulation of the key inflammatory miR-21 (Ando et al. 2013), and in close relevance to the present study, miR-21 has showed consistent and significant down-regulation in B cells of PBC patients from stage I to stage III of PBC (Wang et al. 2017). The biological significance of this finding in a presumably T-cell mediated autoimmune liver diseases and its pathophysiological connotations in AMA positive versus AMA negative patients remains elusive. Moreover, some reports demonstrated evidence for the role of miR-21 in fibrosis (Afonso et al. 2018), but others negated the importance of this microRNA in HCS activation and liver fibrosis (Caviglia et al. 2018).

Similarly to miR-21, miR-150 modulates immune response and is functionally impaired in immune-mediated diseases which frequently co-exists with PBC (Bergman et al. 2016; Chen et al. 2017). MiR-150 shapes the maturation of T cells, B cells, and NK cells via direct downregulation of cMyb, a modulator of hematopoietic cells biology (Bezman et al. 2011; Liu et al. 2013). Inhibition of miR-150 affects antibody production with significantly higher levels of immunoglobulins of various classes (Xiao et al. 2007). Although the expression of cMyb is basically limited to the cells of haematopoietic lineages, cMyb was additionally detected in other cells including hepatic stellate cells (HSC) in which it is critical for these cells activation and proliferation. Moreover, it was suggested that miR-150 regulates activation of HSCs and liver fibrosis, at least in part, via inhibition of cMyb (Venugopal et al. 2010).

Appreciating the roles of miR-21 and miR-150 in modulating immunity and to further address their potential involvement in liver fibrosis and autoagression specifically targeting biliary epithelial cells, we evaluated a comprehensive expression analysis of those microRNAs and their downstream targets in serum, PBMCs and livers of patients with PBC in particular in the context of the presence or absence of disease-specific AMA and the development of liver fibrosis.

## Materials and methods

### Materials

Patients with PBC fulfilling the European Association for the Study of the Liver EASL Clinical Guidelines criteria were recruited from the Liver and Internal Medicine Unit, Medical University of Warsaw (Poland) (European Association for the Study of the Liver 2009). Initially, serum samples from 70 patients with PBC were analyzed. Due to observed significant differences in microRNAs expression between AMA-positive (*n* = 64) and AMA-negative (*n* = 6) patients, further 10 consecutive AMA-negative patients were included in the study, reaching a total of 16. Clinical data on included patients are summarized in Table 1. Liver cirrhosis was diagnosed on the basis of clinical/biochemical and imaging features. Thirty nine patients underwent liver biopsy. The evaluation of a liver biopsy requires the use of routine hematoxylin and eosin (H and E) staining (and occasionally other more specialized stains), to highlight the architecture, and degree of fibrosis. Their fibrosis was assessed according to Batts-Ludwig score (Batts & Ludwig 1995). It contains 5 scoring points reflecting progression of fibrosis. These include: 0 – no fibrosis; 1 – fibrous portal expansion; 2 – rare bridges or septae; 3 – numerous bridges or septae; 4 – cirrhosis.

**Table 1:** Clinical and laboratory characteristics of 80 patients with PBC

Feature	PBC (*n* = 80)	AMA negative (*n* = 16)	AMA positive (*n* = 64)	*p* value ^a^
Age (years)	51.1 ± 8.9	51.1 ± 10.1	51.1 ± 8.7	NS
Gender (F/M)	76/4	15/1	61/3	NS
Cirrhosis (yes/no/unknown)	39/37/4	3/12/1	38/23/3	***p*** **= 0.004**
ALT (IU/l; Normal:< 30)	114 ± 102	98 ± 141	119 ± 89	NS
AST (IU/l; Normal:< 30)	99 ± 83	67 ± 61	108 ± 87	NS
ALP (IU/l; Normal:< 120)	455 ± 286	238 ± 184	516 ± 281	***p*** **= 0.0005**
GGT (IU/l; Normal:< 42)	467 ± 517	230 ± 181	546 ± 555	***p*** **= 0.015**
Bilirubin (mg/dl; Normal< 1.0)	2.3 ± 2.7	1.8 ± 2.4	2.5 ± 2.7	NS

^a^ *p* values: AMA-negative vs AMA-positive

Bold entries have significant values

Serum samples from 19 age- and sex-matched healthy subjects were tested with QUANTA Lite PBC autoantibody Screen IgG/IgA ELISA kit (Inova Diagnostics) in order to exclude PBC-specific autoantibodies.

Liver tissue samples were collected from histologically proven cirrhotic livers of PBC patients (PBC *n* = 21) who underwent liver transplantation. Control liver tissues (*n* = 14) were secured from large margin liver resections of colorectal metastases as previously described (Wunsch et al. 2015).

Peripheral blood mononuclear cells (PBMCs) were freshly isolated from heparinized venous blood samples of PBC patients (*n* = 15) and healthy subjects (*n* = 8).

### RNA extraction and quantification of gene expression

RNA was isolated from the liver tissue and PBMCs with the RNeasy Mini kit (Qiagen). The expression of *RASGRP1*, *CMYB*, *DNMT1* mRNAs and 18S rRNA was measured with Gene Expression Assays (Hs00996734, Hs00920556, Hs00945875, Hs99999901_s1 respectively; Applied Biosystems) by quantitative real-time PCR. The relative quantification of target genes expression was calculated with the 2^-ΔΔCt^ method.

### MicroRNA extraction and quantification

Total RNA was isolated with the miRNeasy Mini Kit (Qiagen) from liver tissues of PBC patients (*n* = 21) and controls (*n* = 14), as well as from PBMCs of PBC patients (*n* = 15) and healthy subjects (*n* = 8). Serum RNA extraction was carried out with miRNeasy Serum/Plasma *Kit* (Qiagen) on sera collected from PBC patients (*n* = 76) and healthy subjects (*n* = 19). Prior to RNA extraction, 200 μl of each serum aliquot was spiked-in with 5 fmol synthetic *C.elegans* microRNA (cel-miR-39-3p; Ambion). cDNA was synthesized with the TaqMan Advanced miRNA *cDNA* Synthesis Kit (Applied Biosystems). The expression of miR-21 and miR-150 was measured with TaqMan® Advanced miRNA Assays (477975_mir and 477918_mir respectively; Applied Biosystems). MiR-191-5p (477952_mir; Applied Biosystems) was used as a endogenous control for liver samples and PBMCs, whereas cel-miR-39 (*478293*_mir*;* Applied Biosystems*)* was an exogenous control for sera samples. The comparative Ct method 2^-ΔΔCt^ was used to calculate the changes in miRNA expression of all samples relative to a non-diseased sample, which was designated as the calibrator.

### Ethics

Written informed consent was obtained from each patient prior to enrolment in the study. The study protocol was approved by the Ethics Committee of Pomeranian Medical University and conforms to the ethical guidelines of the 1975 Declaration of Helsinki (6th revision, 2008).

### Statistics

Data were evaluated as mean ± standard error (SEM) for continuous variables and analyzed using Stat-View-5 Software (SAS Institute, Cary, NC, US) and included ANOVA analysis. Correlation analysis was performed using the Pearson’s correlation method. A *p* value < 0.05 was considered statistically significant.

## Results

### Serum expression of miR-21 and miR-150

**Fig. 1:**
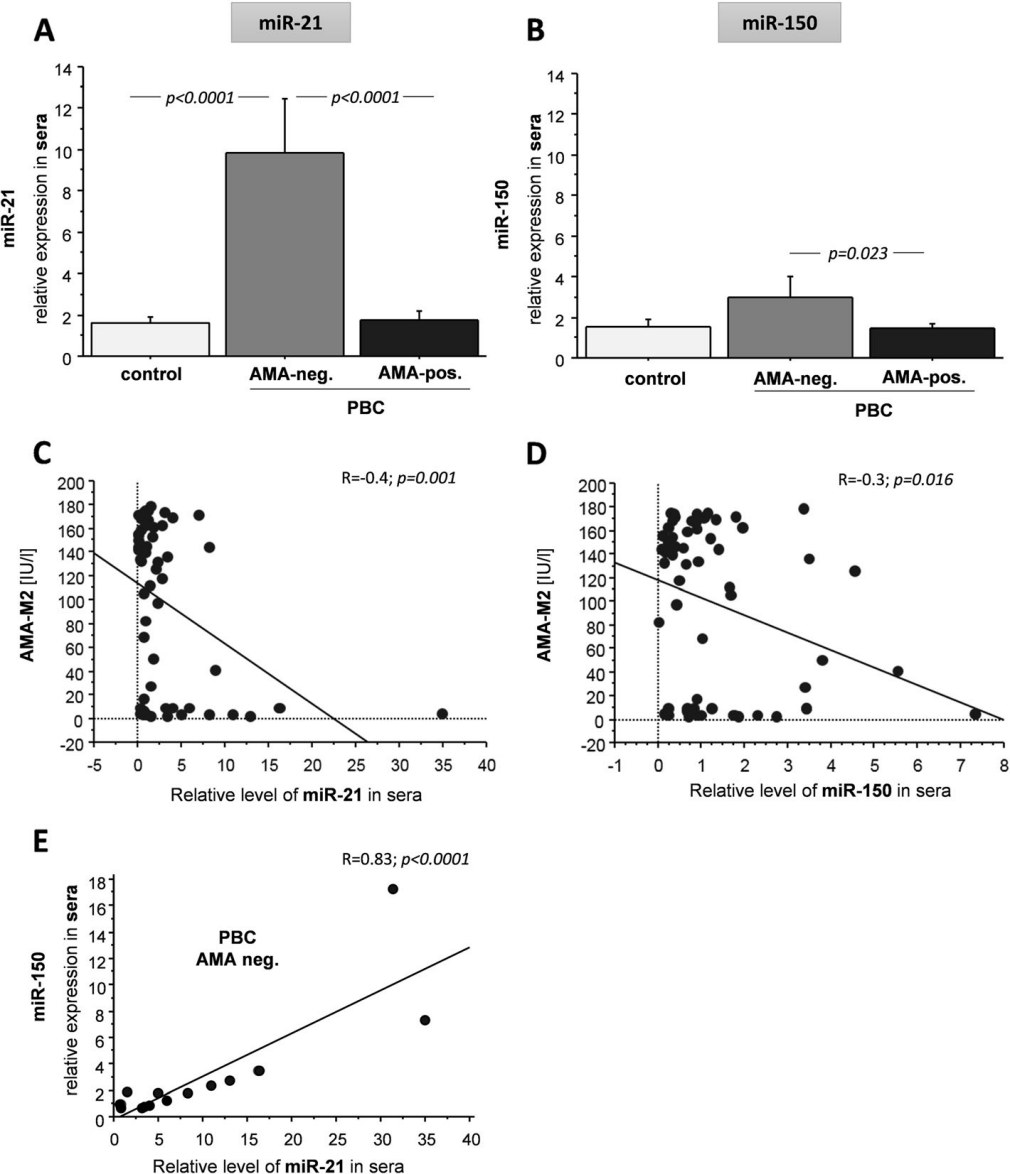
The relative levels of miR-21 and miR-150 in relation to AMA status and AMA-M2 tires in sera of PBC patients. Mir-21(**a**) and miR-150(**b**) levels were significantly higher in patients without AMA antibodies (AMA-negative) than in patients with AMA antibodies (AMA-positive.). Bars indicate the mean ± SEM. There was a strong negative correlation between both miR-21(**c**) and miR-150 (**d**) and AMA-M2 serum tires in all PBC patients. There was a strong positive correlation between the levels of miR-21 and miR-150 in AMA-negative PBC patients (**e**). R-denotes linear association measured by Pearson correlation coefficient test

Neither serum miR-21 nor miR-150 was significantly different between PBC and controls (data not shown). However, expression of miR-21 in AMA-negative patients has shown a 6.5-fold increase compared to controls (*p* < 0.0001) and 5.7-fold compared to AMA-positive PBC patients (*p* < 0.0001) (Fig. 1a-b). Likewise, serum miR-150 levels were higher in AMA-negative patients than AMA-positive PBC patients (1.9-fold increase, *p* = 0.023; Fig. 1b). Moreover, the levels of both micro RNAs negatively correlated with serum AMA titers in AMA-positive PBC patients (*R* = 0.4 for miR-21, *p* = 0.001, and *R* = 0.3 for miR-150, *p* = 0.016; Fig. 1c-d). In AMA-negative, but not AMA-positive group of patients, there was a strong positive correlation between those microRNAs (*R* = 0.83; *p* < 0.0001) (Fig. 1e).

**Fig. 2:**
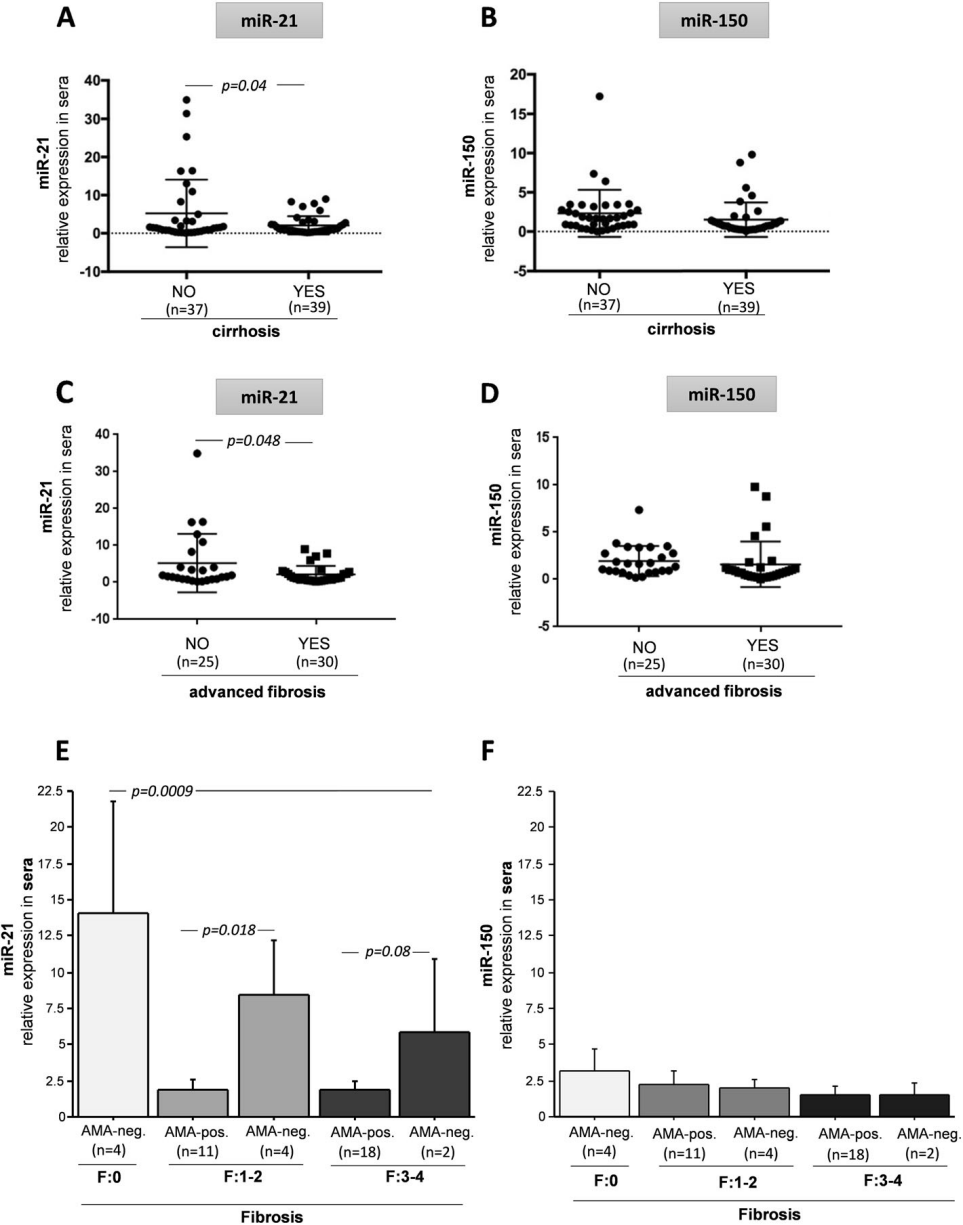
The relationships between the occurrence of cirrhosis, the stage of fibrosis and serum levels of miR-21 or miR-150. The lower expression of miR-21 was observed in PBC patients with cirrhosis in contrast to non-cirrhotic patients (**a**), in regard to miR-150, there was no difference between cirrhotic and non-cirrhotic patients (**b**). PBC patients without advanced fibrosis had the higher level of miR-21(**c**), whereas the level of miR-150 was comparable among all PBC patients independently of fibrosis status (**d**). In contrast to miR-150 (**f**), miR-21 expression changes dependently of fibrosis stages (**e**). Bars indicate the mean ± SEM

Changes in the expression of either miR-21 or miR-150 did not correlate with liver biochemistry features. However, the serum levels of miR-21 was higher in non-cirrhotic patients in comparison to cirrhotic PBC patients (2.6-fold increase, *p =* 0.04; Fig. 2a). Furthermore, in patients without advanced fibrosis (F0-F2) there was a 2.5-fold increase in the level of miR-21 compared to patients with (F3-F4) advanced fibrosis (*p =* 0.048; Fig. 2c). MiR-21 expression was substantially enhanced in serum of patients without fibrosis (F0) in comparison to patients with fibrosis (all AMA-negative, *n* = 4; *p* = 0.0009, Fig. 2e). However, miR-21 expression was enhanced in all AMA-negative patients independently of the stage of liver fibrosis. Accordingly, both in a group of patients with fibrosis stage F1–2 and in a group of patients with fibrosis F3–4, the AMA-negative patients had a higher level of miR-21 in comparison to AMA-positive PBC (4.6-fold, *p =* 0.018, and 2.5-fold *p* = 0.08; respectively, Fig. 2e).

On the contrary, miR-150 expression was not influenced by established cirrhosis (Fig. 2b) or advanced fibrosis (Fig. 2d), and its serum level was not affected by staging of fibrosis (Fig. 2f).

### Levels of miR-21 and miR-150 in liver tissue and in PBMCs of PBC patients

**Fig. 3:**
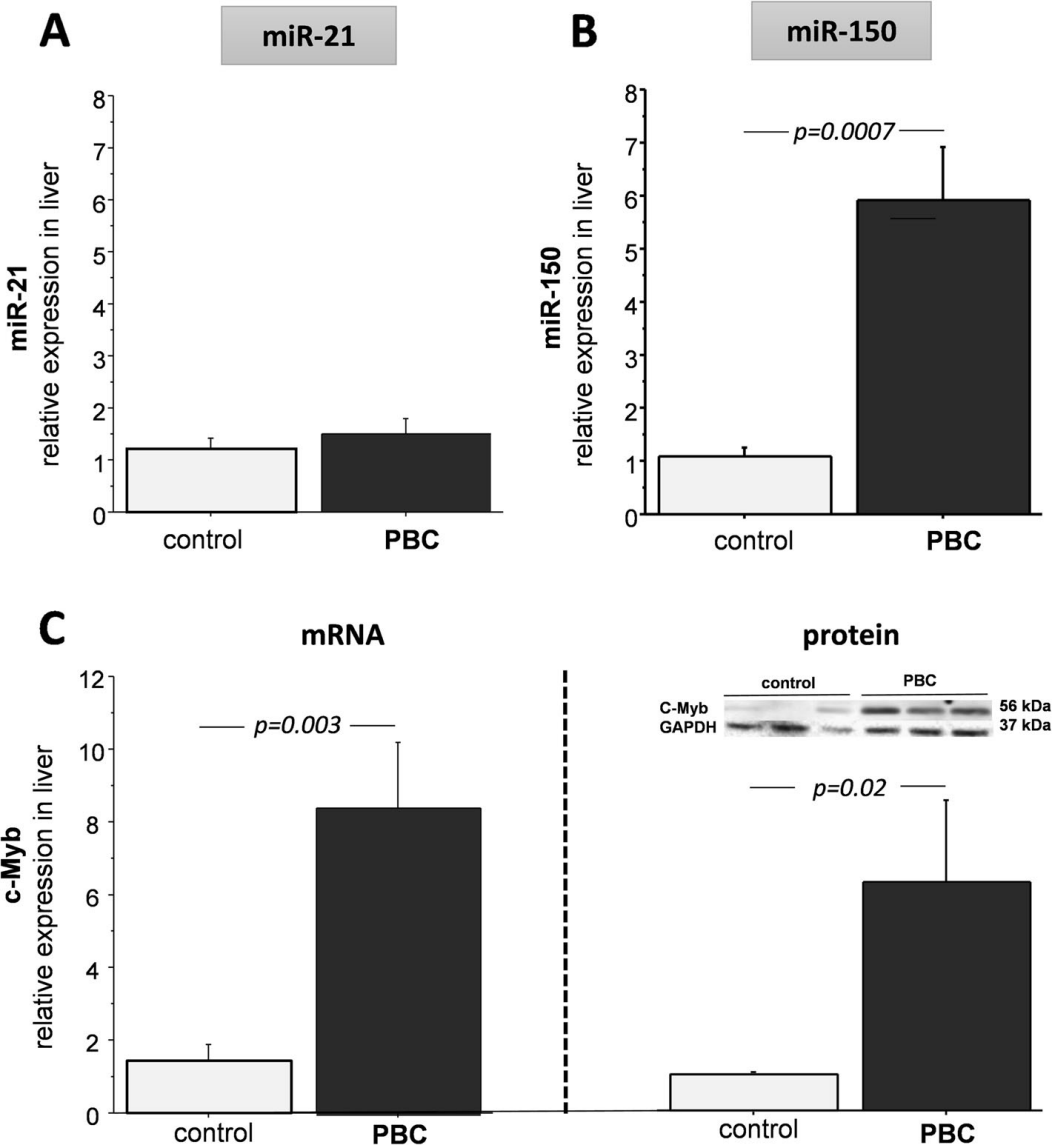
Expression of miR-21, miR-150 and cMyb in cirrhotic livers of patients with PBC**.** There was no significant difference in the level of miR-21 between PBC patients and controls (**a**). The enhanced expression of miR-150 in cirrhotic livers (**b**) was associated with the increased cMyb mRNA and protein levels (**c**). Bars indicate the mean ± SEM

The analysis of microRNA expression with Affymetrix GeneChip miRNA 4.0 Array demonstrated enhanced expression of both miR-21 and miR-150 in livers of patients with PBC (7-fold increase, *p* < 0.0001vs.controls, and 6.1-fold increase, *p* < 0.0001 vs. controls, respectively; data not shown). When real-time PCR (with internal reference i.e. miR-191) analysis was performed, no significant difference was noted in the level of miR-21 between PBC and controls (Fig. 3a). However, for miR-150 the expression of this microRNA was 5.4 times higher in cirrhotic PBC livers than in control tissues (*p* = 0.0007; Fig. 3b). The expression of cMyb, a downstream target of mir-150, was also enhanced in liver tissue of PBC patients (5.9-fold increase of mRNA, *p* = 0.003 vs. controls, and 6-fold increase of its protein level *p* = 0.02 vs controls; Fig. 3c).

**Fig. 4:**
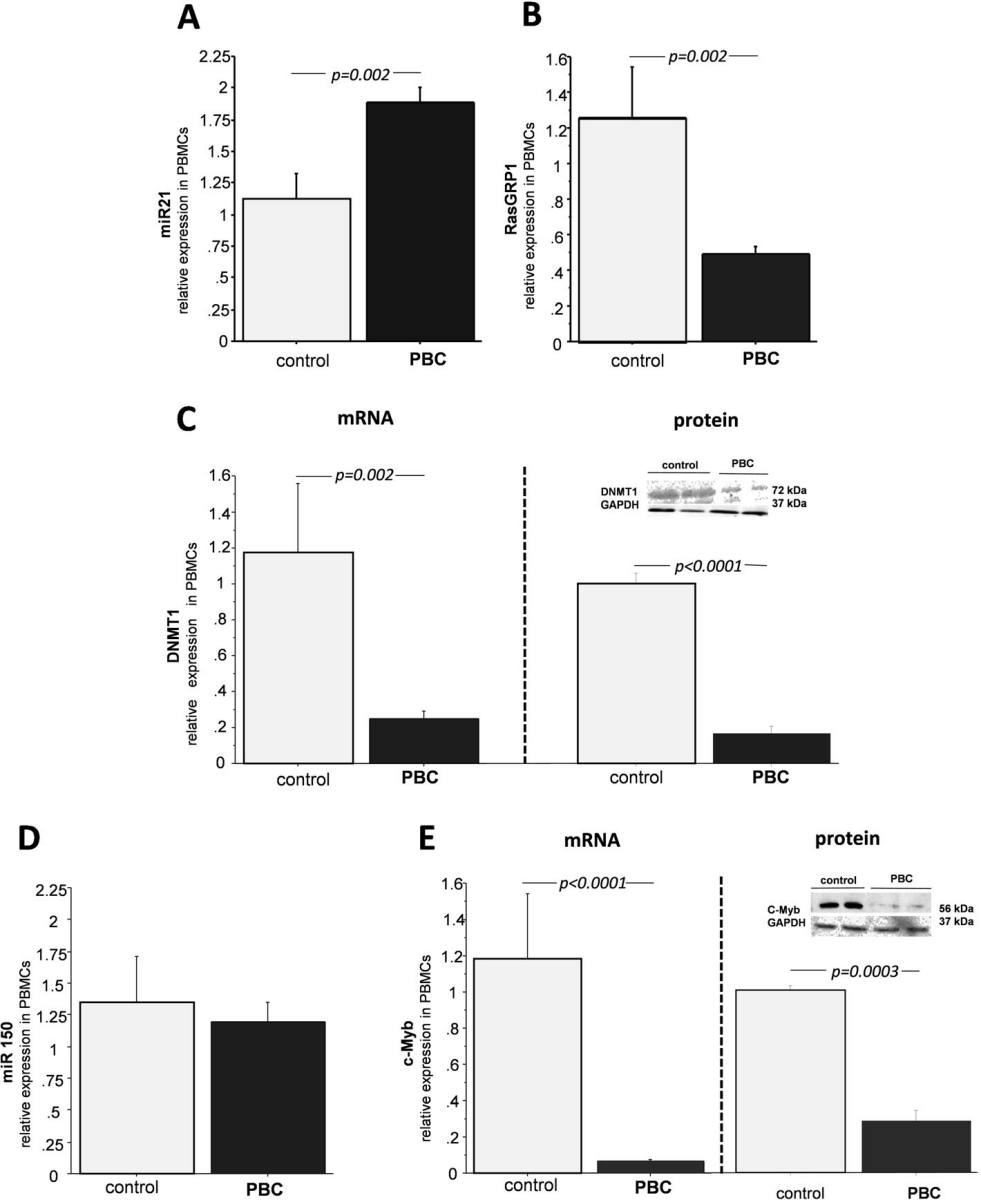
The expressions of miR-21, miR-150 and their downstream target genes in peripheral blood mononuclear cells (PBMCs). MiR-21 expression was increased in PBMCs of patients with PBC in comparison to controls (**a**). The expression of RASGRP1 gene (**b**) and DNMT1(**c**) mRNA and protein levels were reduced in PBMCs of PBC patients. MicroRNA 150 expression was unchanged (**d**),whereas cMyb mRNA and protein levels were significantly decreased in PBMCs of PBC patients (**e**). Bars indicate the mean ± SEM

Next, we assessed the expression of both microRNAs in *PBMCs.* We found that the expression of miR-21 was enhanced 1.7 times in PBC (*p* = 0.002 vs. controls, Fig. 4a), and it was accompanied by downregulation of its downstream target RASGRP1 mRNA (67% reduction; *p* = 0.002 vs. controls; Fig. 4b), as well as the downregulation of DNMT1 (80 and 83% reduction of mRNA and protein level, respectively; *p* = 0.002, and *p* < 0.0001vs.controls, respectively, Fig. 4c). In comparison to controls, the level of miR-150 was unchanged in PBMCs isolated from PBC patients; Fig. 4d) but was accompanied by the decreased expression of cMyb mRNA (90% reduction; *p* < 0.0001 vs. controls Fig. 4e) and protein level (90% reduction; *p* < 0.0001 vs. controls Fig. 4e).

## Discussion

We herein report, for the first time in a collective manner involving, serum, PBMC and tissue analysis, the difference in the levels of miR-21 and miR-150 in sera of patients with PBC and state that this depends on their AMA-status. Furthermore, the alterations of miR-21 level of PBMCs followed by changes of the downstream targets RASGRP1 and DNMT1 underline the importance of mir-21 in immune dysregulation and suggest an important mechanism, which differentiate the induction of the disease in a subgroup of patients who lack AMA. Finally, we demonstrated that the enhanced expression of miR-21 did not directly control the development of liver fibrosis.

Serum levels of miR-21 and miR-150 in AMA-negative patients were increased and the level of each miRNA inversely correlated with AMA antibody titers. The key question raised by these results is whether such findings have a causal or casual relationship in PBC. Reported data in healthy individuals are in favor of the former relationship. In CD4+ T cells from healthy individuals, overexpression of miR-21 promotes autologous B cell differentiation into plasma cells followed by increased IgG production (Stagakis et al. 2011). According to these data, it is reasonable to assume that the increased levels of miR-21 in serum and their over-expression in PBMC and liver of early stage patients with PBC may account for the complete lack of AMA or their appearance at very low titres. If this holds true, then PBC is a disease typically characterized by AMA, in which aberrant over-expression of miR-21 leads to inhibition of autoantigen–specific plasmocyte produced AMA. However, caution must be exercised in raising conclusive expectations because of data in patients with systemic lupus erythematosus (SLE), reporting that specific inhibition of miR-21 in autologous co-cultures of T/B lymphocytes leads to the reduction and not the increase of total IgG, which may also impact autoantibody reduction. Having said that, PBC and SLE infrequently overlap, clearly indicating distinct mechanisms driving the underlying immunopathogenic processes. Another characteristic feature of PBC, namely the increase of IgM but not that of IgG, fits with the mechanistic miR-21 mediated features described by Stagakis et al. (Stagakis et al. 2011). If over-expression of miR-21 is directly or indirectly responsible for AMA negativity, and possibly AMA-negative PBC, the increase of miR-21 would be a feature noted at pre-clinical or early non-fibrotic stages of the disease rather than an epiphenomenon subsequent to tissue destruction in cirrhotic livers. Our findings strikingly demonstrate that this holds true as miR-21 overexpression is an early sign of the disease at fibrotic stages. The fact that fibrotic livers of AMA-negative PBC patients sustain their miR-21 over-expressed status can only be a testimony of the intrinsic feature of this subgroup of patients and the involvement of this miR-21 by both immunological and non-immunological pathways.

Intriguingly, a concomitant increase in miR-150 expression was also observed in sera of AMA-negative patients. The relation between serum level of this microRNA and antibody production was reported previously (Ye et al. 2019). Significantly higher levels of diverse immunoglobulins classes were present in blood of miR-150 knockout mice (Xiao et al. 2007). Along this line, the observed higher level of miR-150 is sera of AMA-negative patients which positively correlate with miR-21 expression might lead to the inhibition of AMA production and secretion.

Though the interplay between those microRNAs in autoimmunity progression is not fully comprehended, lessons can be learned by myasthenia gravis (MG), an autoantibody-mediated neuromuscular disorder (Punga et al. 2015). MicroRNA-21 via inhibition of RASGRP1 gene expression downregulates DNA methyltransferase1 (DNMT1), and methylation-silencing of miR-150 gene by DNMT1 as recently reported (Hoareau-Aveilla et al. 2015). Thus, we speculate that miR-21 may indirectly modulate miR-150 expression by downregulation of RASGRP1 that reduces expression of DNMT1 and hypomethylation may lead to the upregulation of miR-150 gene.

**Fig. 5:**
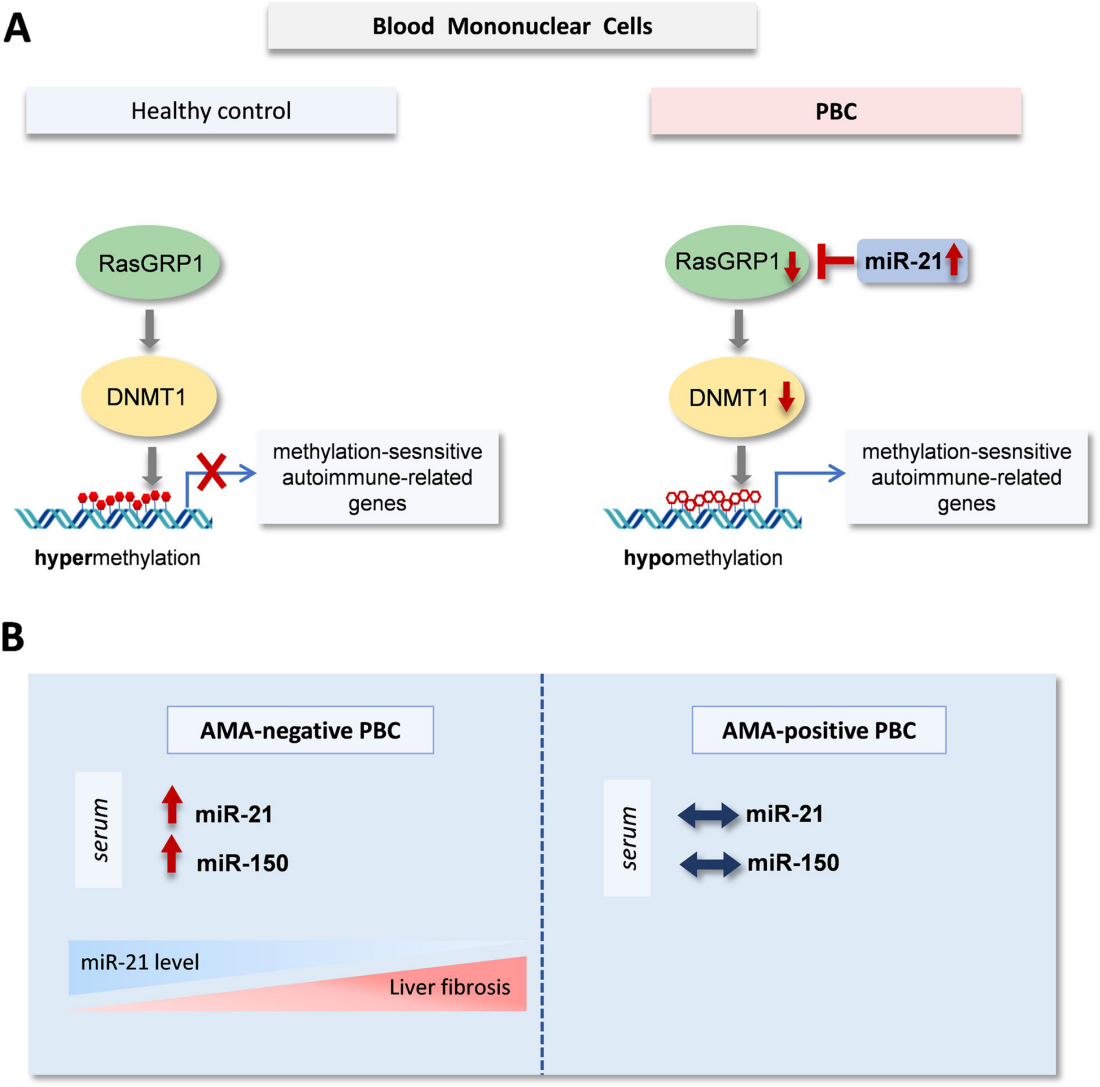
**a.** The increased level of miR-21 in PBMCs leads to the downregulation of RASGRP1 mRNA with the concomitant decrease of DNMT1, triggering immune-driven progression of PBC. **b.** AMA- negative PBC patients are characterized by enhanced expression of miR-21 and miR-150. The elevated levels of serum miR-21 in AMA- negative PBC decreases gradually as liver fibrosis develops

Since the progression of PBC is associated by the constant activation of PBMCs we analyzed the level of miR-21 and miR-150 in those cells. Level of miR-21, but not miR-150, was significantly elevated in PBMCs of PBC patients, and it was accompanied by the reduction of its target gene, RASGRP1. Ras guanyl nucleotide releasing protein 1 (RASGRP1) is expressed in T cells, and to a lesser extent in B and NK cells. Recently, the potential role of RASGRP1 in development of autoimmune diseases was reported. For instance, RASGRP1-dependent downregulation of DNA methyltransferase promotes CD4+ T cell hypomethylation, followed by induction of methylation-sensitive genes which are associated with autoimmunity (Pan et al. 2010). Likewise, in RASGRP1-deficient mice development of CD4 Treg cells in the thymus is severely impaired, and loss-of-function mutations of RASGRP1 was identified in autoimmune diseases (Chen et al. 2014; Mao et al. 2018). Our findings suggest that increased level of miR-21, followed by the decreased level of RASGRP1 mRNA with the concomitant downregulation of DNMT1 in PBMCs, may be associated with immune-driven progression of PBC (Fig. 5).

In cirrhotic PBC livers expression of miR-150 was substantially increased. This can be explained by the fact that in comparison to PBMCs, population of immune cells in livers of PBC patients is extensively enriched by resident CD4^+^ (100-fold increase) and CD8^+^ Tcells (10-fold increase) (Shimoda et al. 1998), which is shaped by miR-150 (Trifari et al. 2013). MiR-150 is also crucial for maturation of NK cells which similarly to T cells, infiltrate liver during PBC progression. NK cells directly attack biliary epithelial cells (BECs), following release of autoantigens, and cryptic epitopes which in turn activate autoreactive T cells when presented by APC (Shimoda et al. 2015). It is well recognized that miR-150 affects growth and maturation of immune cells via downregulation of cMyb (Smith et al. 2015). In this study we observed the considerable increase in cMyb expression in the livers of PBC patients, a finding which was rather unexpected taking into account the above mentioned arguments. We believe that the enhanced expression of cMyb is a result of increased frequency of lymphocytes infiltrating the livers of PBC patients when compared to healthy livers, and such results should be interpreted in the context of number of immune cells that express miR-150.

Whether miR-21 plays a direct role in the progression of fibrosis is not clear. Recent data suggesting that silencing of miR-21 lead to the reduction of liver fibrosis induced by bile duct ligation (BDL) (Afonso et al. 2018), but these findings contrasted emerging data. Caviglia et al., using different experimental models of liver fibrogenesis, showed that miR-21 was not instrumental in activation of hepatic stellate cells (HSCs) or the development of liver fibrosis (Caviglia et al. 2018). Our findings showing that the enhanced serum level of miR-21 in AMA-negative PBC was not related to the stage of liver fibrosis enter the heated debate and are in contrast to reports on the increased level of miR-21 in liver tissue of cirrhotic PBC patients (Afonso et al. 2018). However, direct comparison of the results of the two studies is not possible as the exact stage of liver fibrosis was not fully characterized in their patients while in our study we focused on AMA-negative PBC patients missing from their study. Nevertheless, we still insist, but this remains to be validated by independent studies, that the observed enhanced expression of miR-21 is present only -or largely- at early stages of PBC development when there is the highest activation of lymphocytes, which development and differentiation is highly modulated by miR-21. MicroRNA-21 plays an important role in maintaining effector phase of the T cells and its expression is the highest in effector T cells whereas the lowest in naive T cells (Wu et al. 2007).

## Conclusions

In summary, miR-21 is an ideally placed molecule because of its pleiotropic properties and by default can explain the extent of progression and the switch of pro-fibrotic to fibrotic stages in a combined manner through its implication in hepatocyte/biliary epithelial cell homeostasis and immune regulation. Its over-expression may also explain why a subgroup of PBC patients lack AMA or have diminished titres, miR-150 is interrelated and arguably plays its own role, their close interplay governing several of the key elements in the pathogenesis of PBC, as we have noted by the demonstration of the participation of their downstream regulators RASGRP1 and DNMT1. Joint efforts must be placed in work performed in large prospective longitudinal studies incorporating sera, PBMC and liver tissue, recruited by very early AMA-positive and AMA-negative PBC patients.

## Data Availability

The datasets used and/or analysed during the current study are available from the corresponding author on reasonable request.

## Abbreviations

AMA: Anti-mitochondrial-autoantibodies
DNMT1: DNA-methyltransferase-1
PBC: Primary Biliary Cholangitis
PBMCs: Peripheral blood mononuclear cells
RASGRP1: RAS-guanyl-releasing protein-1
